# Schisandrin B Inhibits LPS‐Induced Endometritis Through Attenuating Ferroptosis via AMPK/PGC1α/Nrf2 Signalling Pathway

**DOI:** 10.1111/jcmm.70281

**Published:** 2024-12-09

**Authors:** Jing Yu, Xuewei Li, Min Zhou, Min Lu, Zheng Ruan, Wenshuang Zou, Shaohui Yu

**Affiliations:** ^1^ Department of Endocrinology Affiliated Hospital of Changchun University of Traditional Chinese Medicine Changchun Jilin China; ^2^ Department of Gynaecology Affiliated Hospital of Changchun University of Traditional Chinese Medicine Changchun Jilin China; ^3^ Department of Traditional Chinese Medicine 964th Hospital Changchun China; ^4^ Department of Gastroenterology The Affiliated Hospital to Changchun University of Chinese Medicine Changchun Jilin People's Republic of China

**Keywords:** endometritis, ferroptosis, inflammation, LPS, *Schisandra* B

## Abstract

Endometritis is one of the common reproductive diseases in human and animal. In recent years, a number of studies have found that *Schisandra* B (Sch B), as a natural Chinese medicine extract, has antioxidant, anti‐inflammatory and other biological activities. Based on the above, in this study, mice were used to conduct an in vivo experiment to investigate the effect and mechanism of Sch B on lipopolysaccharide (LPS)‐induced endometritis. Haematoxylin and eosin (H&E) staining was used to detect the pathological changes of uterine tissue and western blot was used to detect the expression levels of signalling pathways and key genes for ferroptosis. The results showed that Sch B significantly inhibited the pathological injury of uterine tissue, myeloperoxidase (MPO) activity, the activation of NF‐κB pathway and the production of TNF‐α and IL‐1β. Furthermore, Sch B effectively inhibited ferroptosis by inhibiting malondialdehyde (MDA) and iron production and promoting the expression of ferroptosis suppressor genes GPX4 and ferritin. In conclusion, Sch B inhibited LPS‐induced endometritis through alleviating inflammatory response and ferroptosis via AMPK/PGC1α/Nrf2 signalling pathway.

## 1 Introduction

Endometritis is a kind of reproductive disease characterised by local inflammation of the endometrium [1]. Inflammation can cause disorders in the uterine environment, disrupt the oestrus cycle, cause reproductive disorders and eventually lead to long‐term infertility [2]. In severe cases, it can cause systemic infection to death, etc., bringing huge economic losses to the development of the aquaculture industry [3]. There are various causes of endometritis in livestock. Bacterial infection is considered to be the most important cause of endometritis and most of them are bacterial virulence factors lipopolysaccharide (LPS) and lipid teichoic acid (LTA) and so on play a role [4]. In the development of endometritis, LPS can induce the activation of neutrophils, causing the release of inflammatory cytokines, which leads to endometrial damage [5]. A large number of studies have shown that inhibiting inflammatory cytokines has a potential therapeutic effect on endometritis [6]. At present, irrigation, antibiotics, hormones and other methods are commonly used to treat endometritis [7]. There are problems such as unsatisfactory treatment effect, drug resistance and drug residue. It is very important to find new therapeutic drugs. Therefore, screening natural and low‐sideline candidate drugs from traditional Chinese medicine to prevent and treat endometritis can provide solutions for the clinical prevention and treatment of endometritis.

*Schisandra chinensis* is a traditional Chinese medicine with a long history [8]. A large number of studies have proved that it has a good effect on the central nervous system, cardiovascular system, digestive system, immune system, etc., and also has a potential anti‐cancer effect [9]. *Schisandra chinensis* is divided into north *S. chinensis* and south *S. chinensis*. Schisandrin B (Sch B) is the highest content of lignan extracted and isolated from *S. chinensis*. It has been shown that Sch B can inhibit the activity of hepatoma HepG2 cells and protect mice from acute liver failure [10]. A previous study demonstrated that Sch B improves cerebral ischaemia and reduces reperfusion injury in rats through TLR4/NF‐κB signalling pathway inhibition [11]. Furthermore, it has been reported that Sch B mitigates hepatic steatosis and promotes fatty acid oxidation by inducing autophagy through AMPK/mTOR signalling pathway [12]. In other studies, Sch B showed strong anti‐inflammatory and antioxidant activity and has a relieving and protective effect on mouse colitis [13]. However, whether Sch B can treat endometritis remains unclear. Therefore, this study will explore the role of Sch B on endometritis and clarify the mechanism.

## 2 Materials and Methods

### 2.1 Reagents

LPS ( *Escherichia coli* 055: B5) and Sch B (purity > 98%) were purchased from Sigma (MO, USA). ELISA kits were purchased from BioLegend (CA, USA). Protein detection kit purchased from Thermo Company. Antibodies were purchased from CST (CA, USA). MPO kit was purchased from Nanjing Jiancheng (Nanjing, China).

### 2.2 Experimental Design and Grouping

Female C57 mice were purchased from the Experimental Animal Center of Bethune College of Medicine of Jilin University. The mice were adapted to the environment for 1 week. They freely ate conventional diets and drank pure water. All animal experiments are conducted in accordance with animal welfare and ethical standards of Jilin University. Sixty mice were randomly divided into five groups with 12 mice in each group: control group, LPS group and LPS + Sch B (20, 40 and 80 mg/kg) groups. Sch B was administered by intraperitoneal injection 1 h before LPS treatment. The doses of Sch B used in this study were based on previous study [14]. LPS‐induced endometritis model was established by given equal amounts of LPS (1 mg/kg) on each side of the uterus [15]. Twenty‐four hours after LPS treatment, uterine tissues from each group were harvested and immersed in 4% paraformaldehyde for haematoxylin and eosin (H&E) staining. The remaining tissues were stored at −80°C for subsequent study.

### 2.3 H&E Staining

Uterine tissue samples of each group were fixed in 4% paraformaldehyde for 48 h. The sample was embedded in paraffin wax and cut into 5 μm slices. After defatting, staining with haematoxylin and eosin was performed under an optical microscope for histological evaluation.

### 2.4 MPO, MDA, Iron and GSH Assay

Uterine tissue is taken and homogenised with reaction buffer on ice. The detection method of MPO activity, MDA, iron and GSH concentrations were performed according to the kit instructions.

### 2.5 ELISA Assay

ELISA kit was used to detect inflammatory cytokines and the specific operation was carried out according to the manufacturer's instruction. The uterus tissue was transferred to a homogeniser on ice and pre‐cooled PBS (mass/volume ratio of 1∶9) was added to prepare the tissue into a homogeniser. Centrifuge the homogenate at 4°C, 12,000 r/min for 10 min. The lipid layer was removed and the intermediate supernatant was collected for detection. The light absorption values of the samples at 450 and 570 nm were detected by enzyme‐labelled apparatus.

### 2.6 Western Blot Analysis

The total protein of uterine tissue was extracted with the extraction kit and the protein concentration was determined with BCA protein detection kit to prepare the sample. The separation glue and concentrate glue were prepared, 12% SDS‐PAGE was performed and then the protein samples were transferred to PVDF membrane and sealed with 5% skim milk at room temperature for 2 h. After cleaning, the primary (1:1000) and secondary antibodies (1:5000) were incubated successively and the results were observed after ECL reagent was added.

### 2.7 Statistical Analysis

The data analysis was performed using GraphPad Prism 7.02 Software. Differences between mean values of normally distributed data were analysed using one‐way analysis of variance (ANOVA) multiple comparisons. *p* < 0.05 was considered statistically significant.

## 3 Results

### 3.1 The Effect of Sch B on LPS‐Induced Endometrial Pathology

**FIGURE 1:**
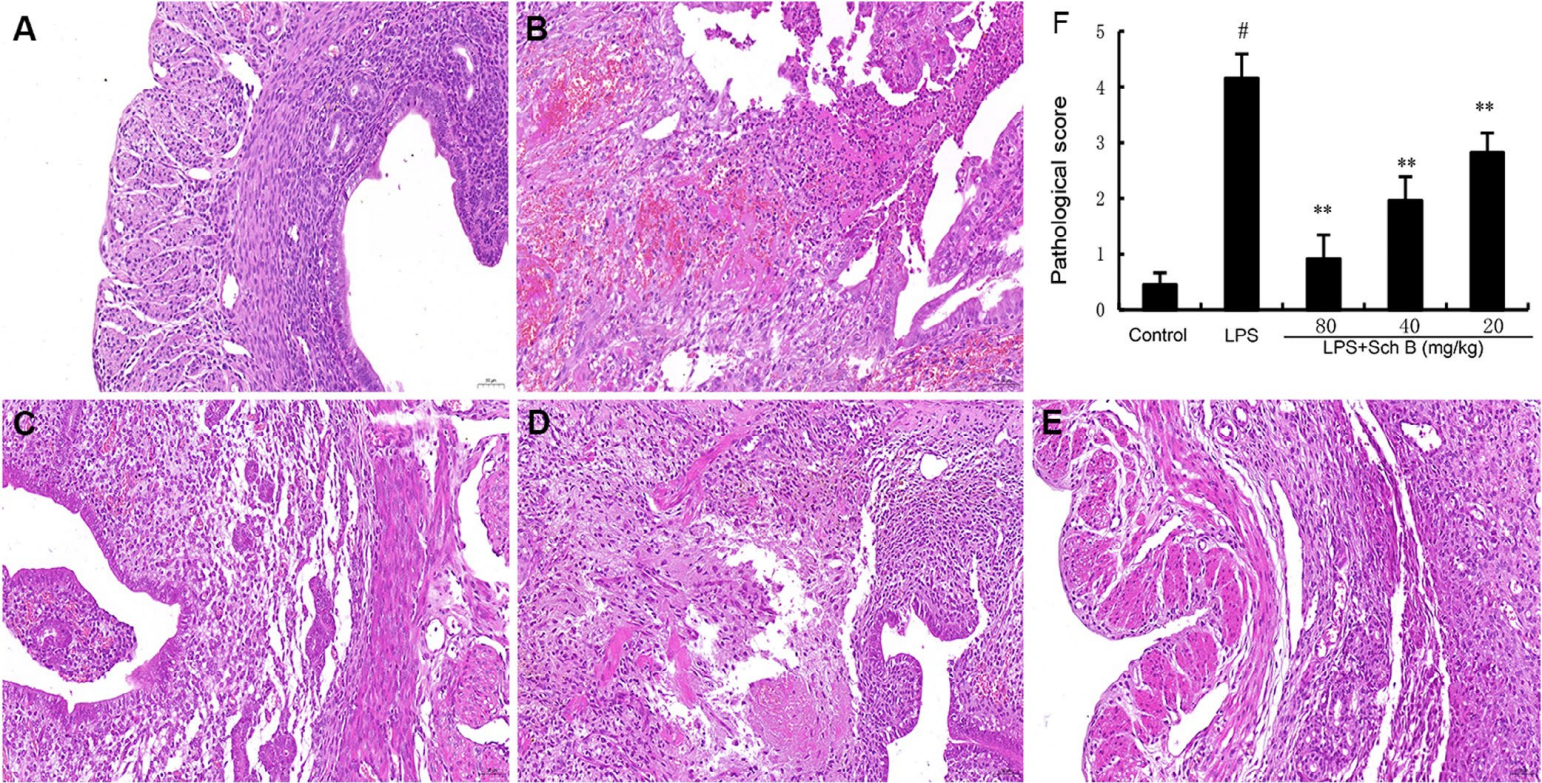
Effects of Sch B on LPS‐induced uterine histopathological changes. Histopathologic sections of uterine tissues (H&E, ×100). (A) Control, (B) LPS group and (C, D, E) Sch B (20, 40 and 80 mg/kg) + LPS groups. (F) Pathological score of uterine tissues. H&E, haematoxylin and eosin; LPS, lipopolysaccharide; Sch B, Schisandra B. #p < 0.01 is significantly different from control group; **p < 0.01 are significantly different from LPS group.

The uterine tissue of mice in the control group was normal with no histopathological changes (Figure 1A). The uterine tissue of mice in the LPS group showed oedema and obvious neutrophil infiltration (Figure 1B). However, SchB treatment significantly weakened the LPS‐induced pathological injury of the uterus and the infiltration of inflammatory cells (Figure 1C–E).

### 3.2 The Effects of Sch B on MPO Activity in LPS‐Induced Uterine Tissue

**FIGURE 2:**
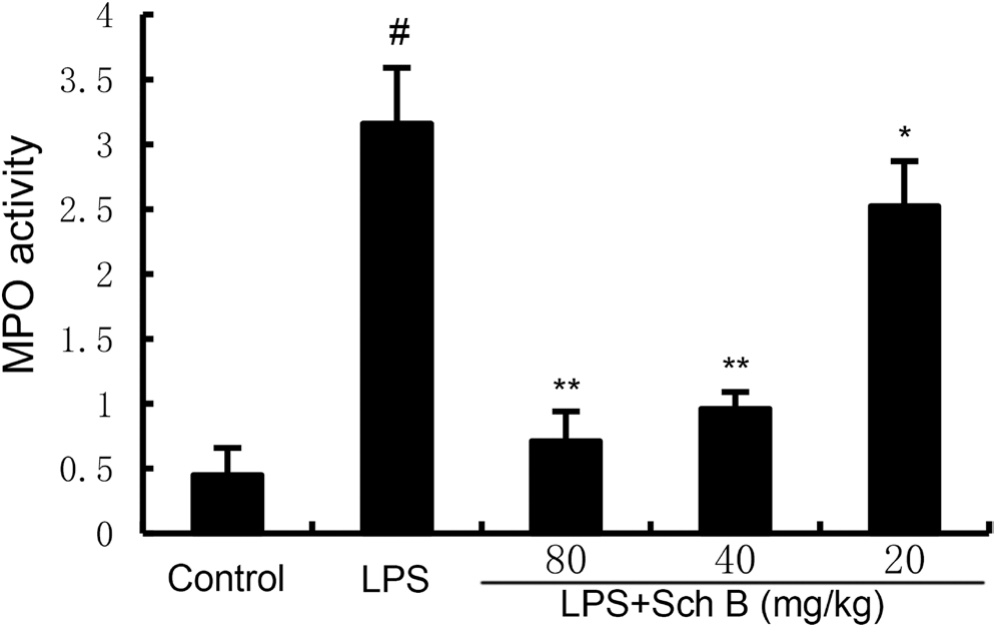
Effect of Sch B on MPO activity. The values presented are the mean ± SD. #p < 0.01 is significantly different from control group; **p < 0.01 are significantly different from LPS group. MPO, myeloperoxidase; Sch B, Schisandra B.

MPO activity can directly reflect the number of neutrophils. As shown in Figure 2, MPO activity in uterine tissue of mice in LPS group was significantly higher than that in control group (*p* < 0.05). Compared with LPS group, MPO activity in uterine tissue of mice pretreated with SchB were significantly decreased (*p* < 0.05).

### 3.3 The Effects of Sch B on the Expression of LPS‐Induced Inflammatory Cytokines

**FIGURE 3:**
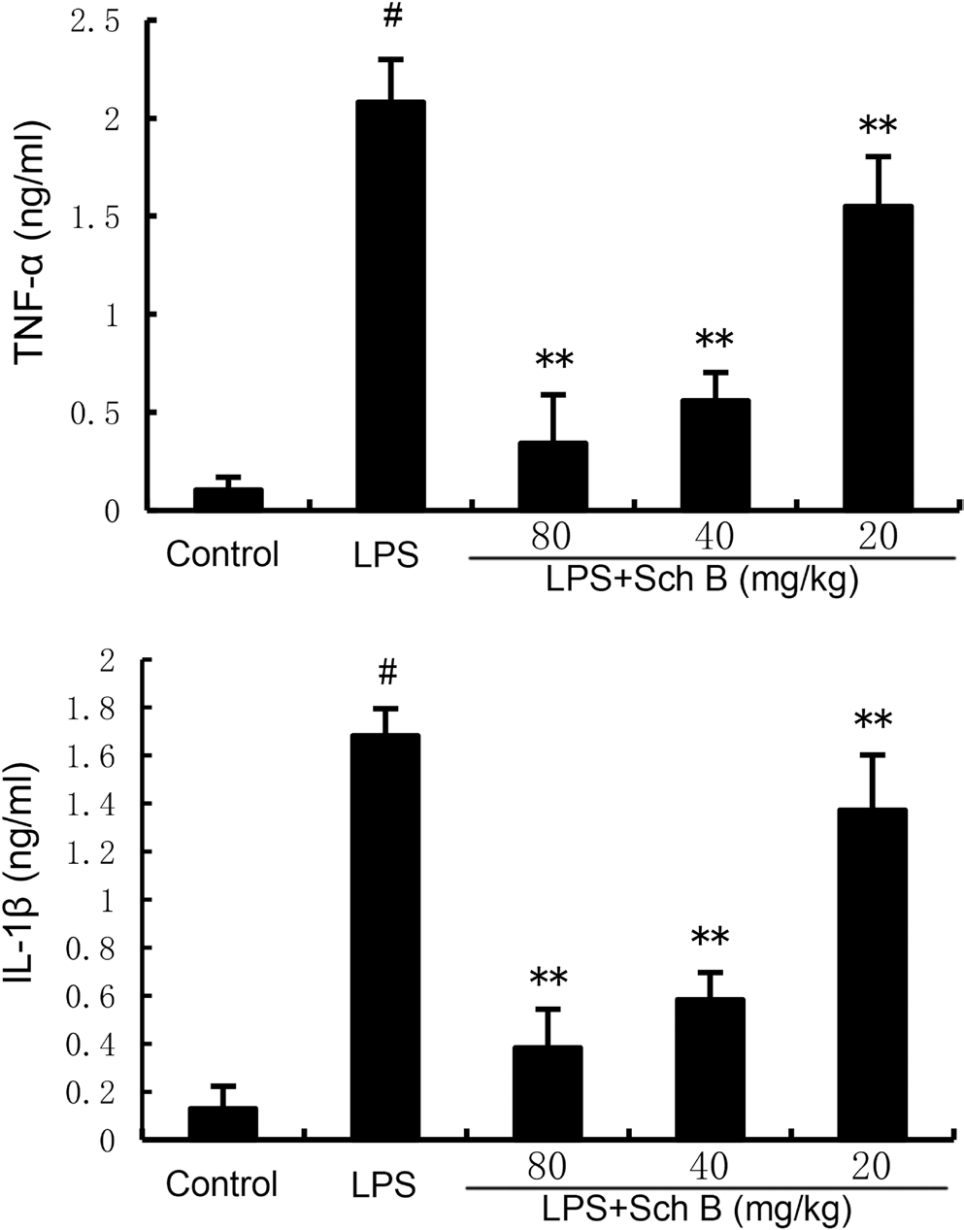
Effect of Sch B on inflammatory cytokine production. The values presented are the mean ± SD. ^#^ *p* < 0.01 is significantly different from control group; **p < 0.01 are significantly different from LPS group. LPS, lipopolysaccharide; Sch B, Schisandra B.

Inflammatory cytokines can reflect the degree of inflammation. As shown in Figure 3, TNF‐α and IL‐1β production in uterine tissue of mice in LPS group was significantly higher than that in control group (*p* < 0.05). Compared with LPS group, TNF‐α and IL‐1β production in uterine tissue of mice pretreated with Sch B were significantly decreased (*p* < 0.05).

### 3.4 The Effect of Sch B on NF‐κB Signalling Pathway

**FIGURE 4:**
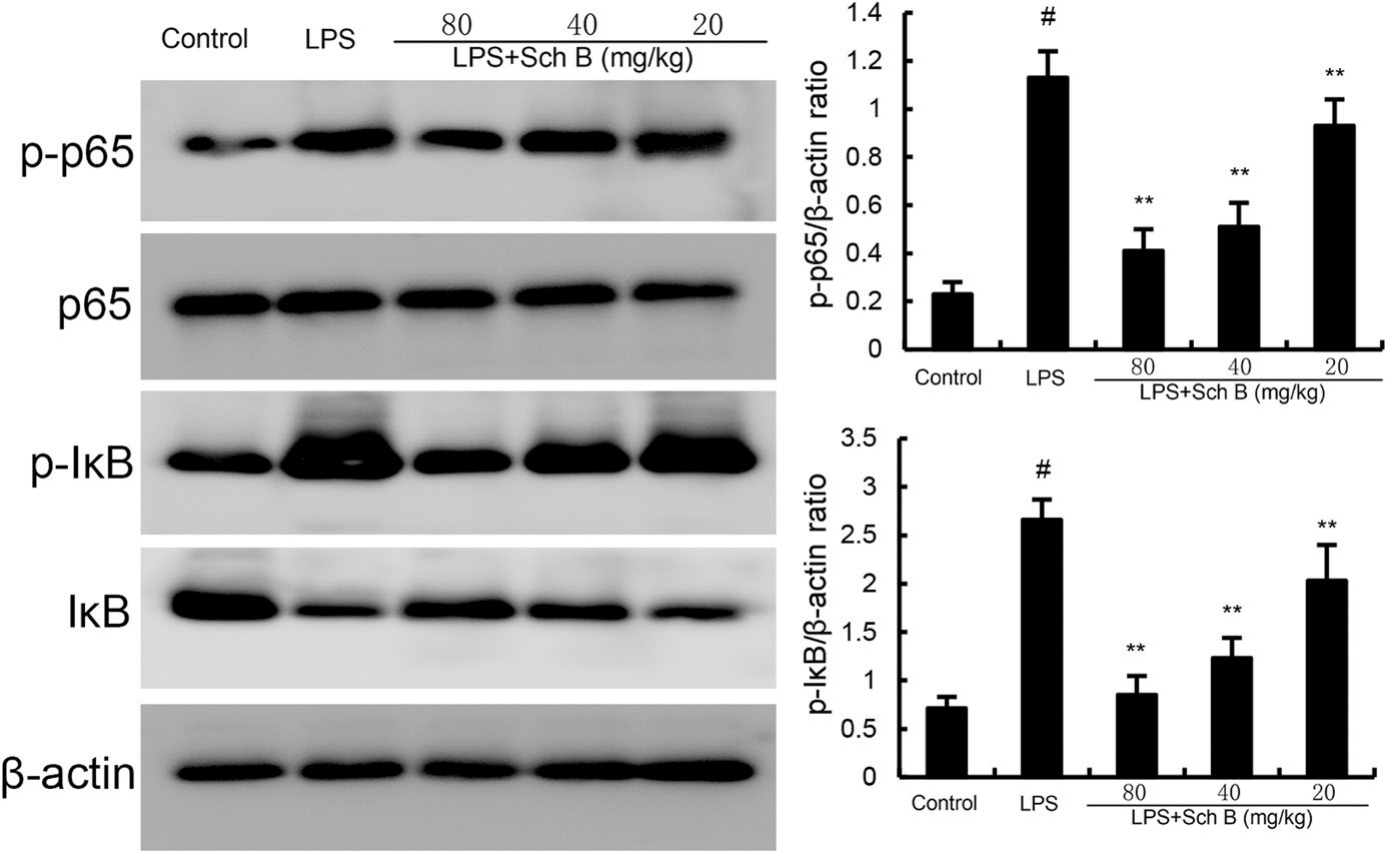
Effect of Sch B on NF‐κB activation in uterine gland. The values presented are the mean ± SD. ^#^ *p* < 0.01 is significantly different from control group; **p < 0.01 are significantly different from LPS group. LPS, lipopolysaccharide; Sch B, Schisandra B.

NF‐κB activation was tested to elucidate the anti‐inflammation mechanism of Sch B. As shown in Figure 4, phosphorylation of IκB and NF‐κB p65 in uterine tissue of mice treated with LPS was upregulated compared with the control group. The phosphorylation levels of IκB and NF‐κB p65 in uterine tissue of mice treated with SchB were significantly reduced compared with those treated with LPS (Figure 4).

### 3.5 The Effects of Sch B on Ferroptosis in LPS‐Induced Uterine Tissue

**FIGURE 5:**
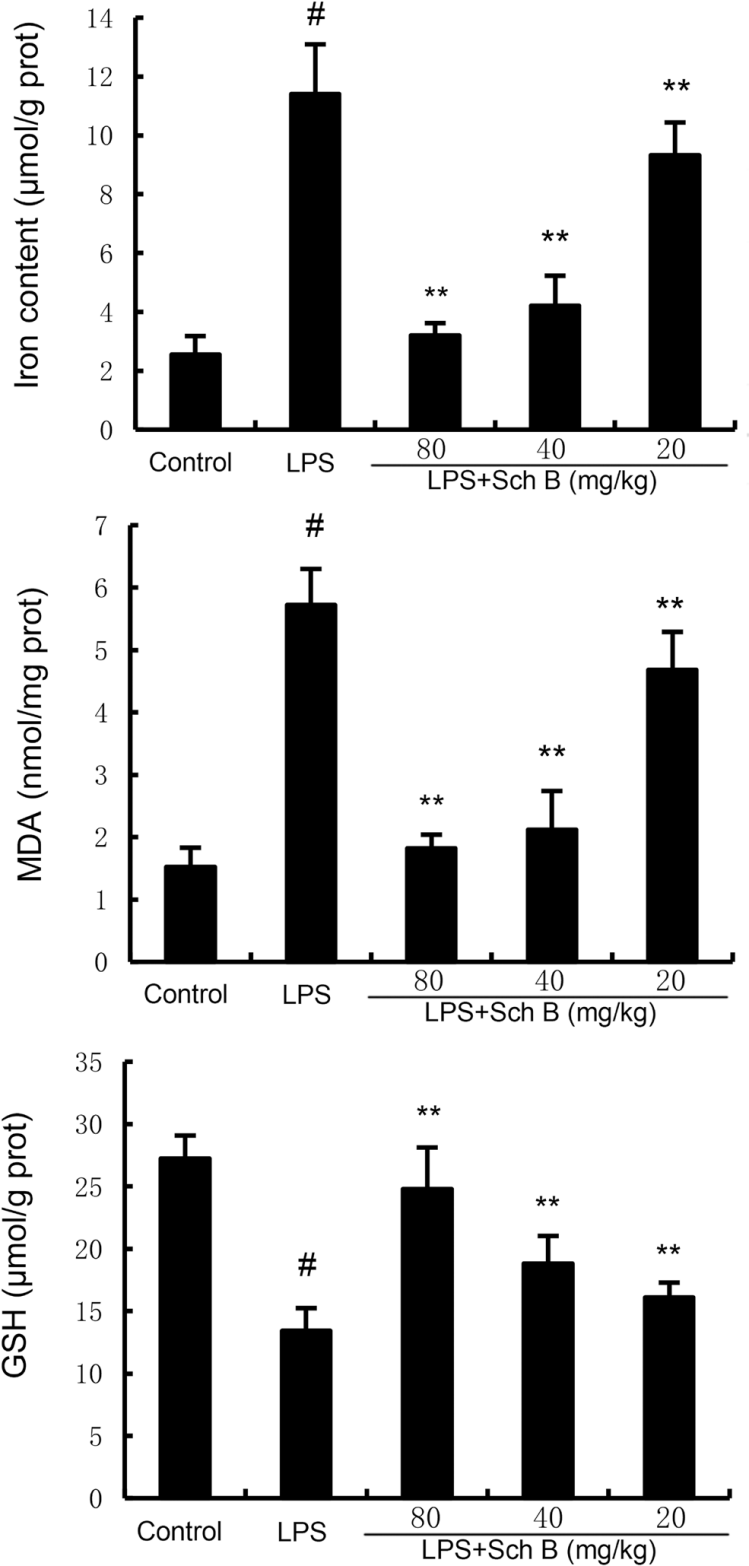
Effect of Sch B on MDA, iron and GSH production. The values presented are the mean ± SD. ^#^ *p* < 0.01 is significantly different from control group; **p < 0.01 are significantly different from LPS group. GSH, synthesis of glutathione; LPS, lipopolysaccharide; MDA, malondialdehyde; Sch B, Schisandra B.

**FIGURE 6:**
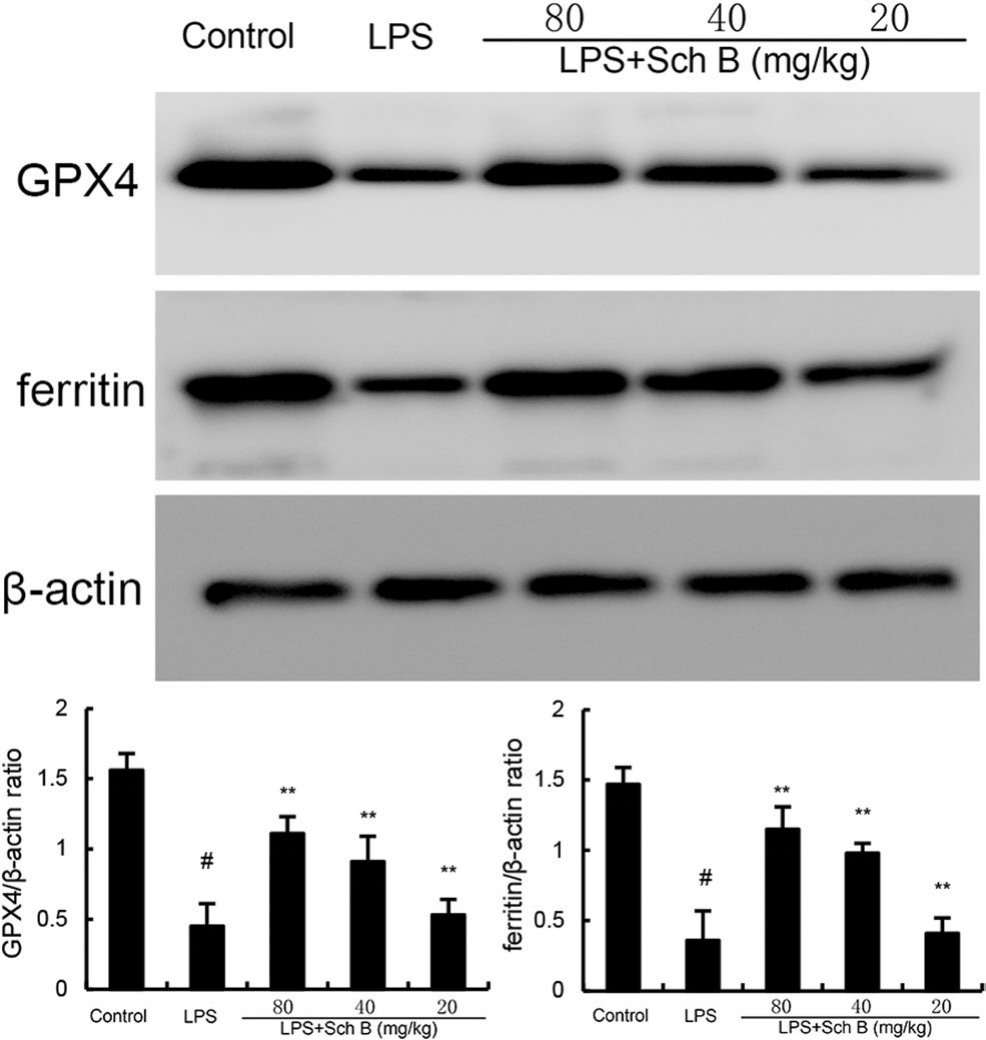
Effect of Sch B on GPX4 and ferritin expression. The values presented are the mean ± SD. ^#^ *p* < 0.01 is significantly different from control group. **p < 0.01 are significantly different from LPS group. GPX4, glutathione peroxidase 4; Sch B, Schisandra B.

As shown in Figures 5 and 6, MDA and iron levels in uterine tissue of mice in LPS group was significantly higher than that in control group (*p* < 0.05). Compared with LPS group, MDA and iron levels in uterine tissue of mice pretreated with Sch B were significantly decreased (*p* < 0.05). Meanwhile, GSH concentration, GPX4 and ferritin expression were decreased by LPS treatment. These factors were upregulated by Sch B treatment (Figures 5 and 6).

### 3.6 Effects of Sch B on AMPK/PGC1α/Nrf2 Signalling Pathway

**FIGURE 7:**
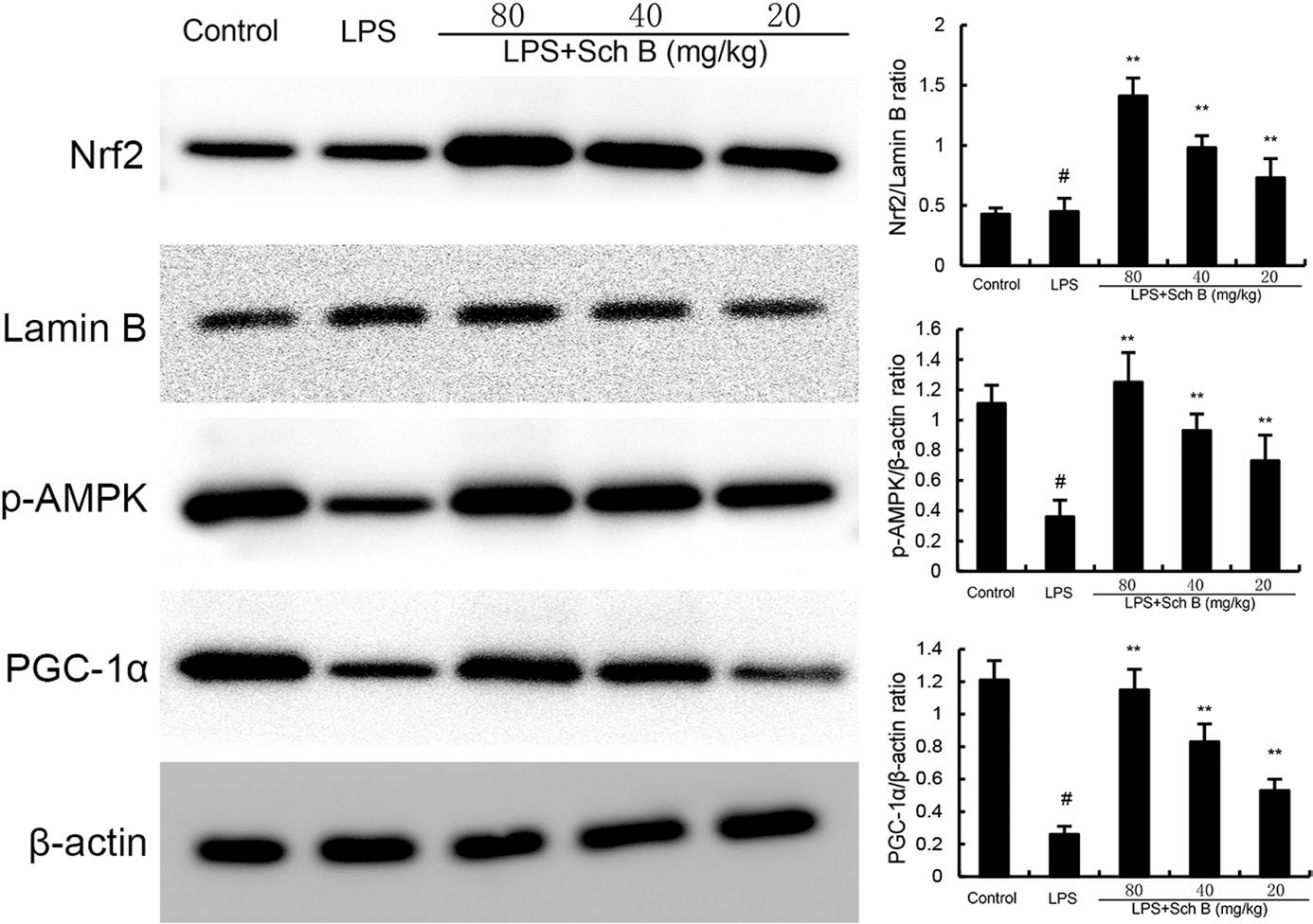
Effect of Sch B on AMPKα, Nrf2 and PGC‐1α expression. The values presented are the mean ± SD. ^#^ *p* < 0.01 is significantly different from control group. **p < 0.01 are significantly different from LPS group. LPS, lipopolysaccharide; Sch B, Schisandra B.

As shown in Figure 7, the expression of phosphorylated AMPK, Nrf2 and PGC‐1α in uterine tissue of mice in LPS group was significantly lower than that in control group (*p* < 0.05). Compared with LPS group, the expression of phosphorylated AMPK, Nrf2 and PGC‐1α were significantly increased by Sch B treatment (*p* < 0.05) (Figure 7).

## 4 Discussion

LPS is the main component of the cell wall of Gram‐negative bacteria and the main causative factor of Gram‐negative bacterial infection, which is the main cause of endometritis [16]. Sch B is the highest content of biphenycloctene lignans in Chinese traditional Chinese schisandrin, which has various physiological effects such as anti‐asthma, anti‐tumour and anti‐inflammatory roles [17]. In this study, H&E staining, western blot, ELISA and other experimental methods were used to explore the regulatory effects of Sch B on LPS‐induced endometritis in mice.

Endometritis is a common multiple disease in dairy cows, which greatly affects the reproductive performance and milk yield of dairy cows [18]. Endometrial inflammation causes damage to the endometrial tissue and affects the normal physiological function of the uterus, thus reducing the pregnancy rate of cows, prolonging the calving interval and even being eliminated [19]. Inflammation plays a crucial role in the development of endometritis [20]. Neutrophils are major cells that release various inflammatory cytokines, such as TNF‐α, IL‐1β and IL‐6. These inflammatory factors are associated with endometrial damage [21]. In this study, inflammatory mediators (i.e., TNF‐α and IL‐1β) were significantly enhanced after LPS treatment. However, Sch B treatment reversed these changes in the uterus and histological examination demonstrated that the reduction of inflammatory mediators was accompanied by the repair of uterine tissue, suggesting that Sch B has a protective effect against endometritis by inhibiting the inflammatory response. The NF‐κB signalling pathway is involved in the regulation of genes that mediate inflammation [22]. Previous studies demonstrated that preventing NF‐κB activation could suppress endometritis [23]. The data indicated that Sch B could significantly inhibit the activity of NF‐κB pathway, indicating the anti‐inflammatory mechanism may be through suppressing NF‐κB pathway.

Ferroptosis is a special form of iron dependent regulatory cell death mainly caused by the accumulation of lipid peroxidation and its occurrence and development are closely related to Nrf2 signalling pathway [24]. The main feature of ferroptosis is to inhibit the synthesis of glutathione (GSH) and the decrease of the activity of glutathione peroxidase 4 (GPX4) [25]. GPX4 is the only known enzyme capable of reducing phospholipid hydroperoxides, inhibiting ferroptosis by using GSH to convert toxic phospholipid hydroperoxides to non‐toxic lipols [26]. Studies have found that AMPK/PGC1α/Nrf2 signalling pathway could regulate ferroptosis [27]. This signalling pathway can also improve the activity of GPX4 by maintaining the balance of iron ions in cells, and prevent ferroptosis in cells against stressed environments. In this study, key indicators of ferroptosis and AMPK/PGC1α/Nrf2 pathway in LPS‐induced mouse endometritis were analysed. The results indicated that GPX4 activity was decreased and AMPK/PGC1α/Nrf2 signalling pathway was inhibited in endometrial tissues in LPS group. After Sch B treatment, GPX4 activity and AMPK/PGC1α/Nrf2 signalling pathway expression were significantly enhanced and ferroptosis in endometrial tissues was alleviated.

In summary, Sch B can reduce the pathological damage of mice endometritis induced by LPS. The mechanism was through inhibiting ferroptosis via regulating AMPK/PGC1α/Nrf2 signalling pathway.

## Author Contributions

**Jing Yu:** data curation (equal), formal analysis (equal), investigation (equal), methodology (equal), resources (equal), software (equal), writing – original draft (equal). **Xuewei Li:** data curation (equal), investigation (equal), methodology (equal), supervision (equal), validation (equal). **Min Zhou:** data curation (equal), formal analysis (equal), investigation (equal), methodology (equal), supervision (equal), validation (equal). **Min Lu:** investigation (equal), methodology (equal), supervision (equal), validation (equal). **Zheng Ruan:** formal analysis (equal), investigation (equal), methodology (equal), resources (equal), validation (equal). **Wenshuang Zou:** conceptualization (equal), investigation (equal), resources (equal), software (equal), visualization (equal). **Shaohui Yu:** conceptualization (equal), funding acquisition (equal), resources (equal), software (equal), validation (equal), visualization (equal), writing – review and editing (equal).

## Conflicts of Interest

The authors declare no conflicts of interest.

## Data Availability

Data will availability when request.

## References

[1] E. Puente , L. Alonso , A. S. Lagana , F. Ghezzi , J. Casarin , and J. Carugno , “Chronic Endometritis: Old Problem, Novel Insights and Future Challenges,” International Journal of Fertility & Sterility 13 (2020): 250–256. 31710184 10.22074/ijfs.2020.5779 PMC6875860

[2] G. Weiss , L. T. Goldsmith , R. N. Taylor , D. Bellet , and H. S. Taylor , “Inflammation in Reproductive Disorders,” Reproductive Sciences 16 (2009): 216–229. 19208790 10.1177/1933719108330087 PMC3107847

[3] I. F. Canisso , J. Stewart , and M. A. C. da Silva , “Endometritis Managing Persistent Post‐Breeding Endometritis,” Veterinary Clinics: Equine Practice 32 (2016): 465–480. 27810036 10.1016/j.cveq.2016.08.004

[4] J. Ravel , I. Moreno , and C. Simon , “Bacterial Vaginosis and Its Association With Infertility, Endometritis, and Pelvic Inflammatory Disease,” American Journal of Obstetrics and Gynecology 224 (2021): 251–257. 33091407 10.1016/j.ajog.2020.10.019

[5] M. L. Turner , G. D. Healey , and I. M. Sheldon , “Immunity and Inflammation in the Uterus,” Reproduction in Domestic Animals 47 (2012): 402–409. 22827398 10.1111/j.1439-0531.2012.02104.x

[6] R. Li , T. Maimai , H. M. Yao , et al., “Protective Effects of Polydatin on LPS‐Induced Endometritis in Mice,” Microbial Pathogenesis 137 (2019): 103720. 31494302 10.1016/j.micpath.2019.103720

[7] K. Kitaya and T. Ishikawa , “Chronic Endometritis: Simple Can Be Harder Than Complex?,” Fertility and Sterility 115 (2021): 1443–1444. 33892957 10.1016/j.fertnstert.2021.03.023

[8] Z. J. Li , X. He , F. Liu , J. Wang , and J. Feng , “A Review of Polysaccharides From *Schisandra chinensis* and *Schisandra sphenanthera*: Properties, Functions and Applications,” Carbohydrate Polymers 184 (2018): 178–190. 29352909 10.1016/j.carbpol.2017.12.058

[9] Q. Lai , J. B. Wei , M. Mahmoodurrahman , et al., “Pharmacokinetic and Nephroprotective Benefits of Using Extracts in a Cyclosporine A‐Based Immune‐Suppressive Regime,” Drug Design Development and Therapy 9 (2015): 4997–5018. 26355803 10.2147/DDDT.S89876 PMC4560515

[10] Y. Zhang , Z. W. Zhou , H. Jin , et al., “Schisandrin B Inhibits Cell Growth and Induces Cellular Apoptosis and Autophagy in Mouse Hepatocytes and Macrophages: Implications for Its Hepatotoxicity,” Drug Design Development and Therapy 9 (2015): 2001–2027. 25926716 10.2147/DDDT.S77071 PMC4403607

[11] X. J. Fan , K. Elkin , Y. W. Shi , et al., “Schisandrin B Improves Cerebral Ischemia and Reduces Reperfusion Injury in Rats Through TLR4/NF‐κB Signaling Pathway Inhibition,” Neurological Research 42 (2020): 693–702. 32657248 10.1080/01616412.2020.1782079

[12] L. S. Yan , S. F. Zhang , G. Luo , et al., “Schisandrin B Mitigates Hepatic Steatosis and Promotes Fatty Acid Oxidation by Inducing Autophagy Through AMPK/mTOR Signaling Pathway,” Metabolism 131 (2022): 155200. 35405150 10.1016/j.metabol.2022.155200

[13] W. D. Li , Y. Liu , Z. Wang , T. Yu , Q. Lu , and H. Chen , “Suppression of MAPK and NF‐κB Pathways by Schisandrin B Contributes to Attenuation of DSS‐Induced Mice Model of Inflammatory Bowel Disease,” Die Pharmazie 70 (2015): 598–603. 26492645

[14] R. Jia , H. Zhang , Z. Yang , et al., “Protective Effects of Schisandrin B on Cigarette Smoke‐Induced Airway Injury in Mice Through Nrf2 Pathway,” International Immunopharmacology 53 (2017): 11–16. 29031142 10.1016/j.intimp.2017.09.030

[15] W. L. Zhao , J. R. Wang , Y. Li , and C. Ye , “Citral Protects Against LPS‐Induced Endometritis by Inhibiting Ferroptosis Through Activating Nrf2 Signaling Pathway,” Inflammopharmacology 31 (2023): 1551–1558. 37010717 10.1007/s10787-023-01211-2

[16] L. C. Carneiro , J. G. Cronin , and I. M. Sheldon , “Mechanisms Linking Bacterial Infections of the Bovine Endometrium to Disease and Infertility,” Reproductive Biology 16 (2016): 1–7. 26952747 10.1016/j.repbio.2015.12.002

[17] R. Checker , R. S. Patwardhan , D. Sharma , et al., “Schisandrin B Exhibits Anti‐Inflammatory Activity Through Modulation of the Redox‐Sensitive Transcription Factors Nrf2 and NF‐κB,” Free Radical Biology & Medicine 53 (2012): 1421–1430. 22917978 10.1016/j.freeradbiomed.2012.08.006

[18] S. J. LeBlanc , T. F. Duffield , K. E. Leslie , et al., “Defining and Diagnosing Postpartum Clinical Endometritis and Its Impact on Reproductive Performance in Dairy Cows,” Journal of Dairy Science 85 (2002): 2223–2236. 12362455 10.3168/jds.S0022-0302(02)74302-6

[19] I. M. Sheldon , J. Cronin , L. Goetze , G. Donofrio , and H. J. Schuberth , “Defining Postpartum Uterine Disease and the Mechanisms of Infection and Immunity in the Female Reproductive Tract in Cattle,” Biology of Reproduction 81 (2009): 1025–1032. 19439727 10.1095/biolreprod.109.077370 PMC2784443

[20] V. A. Kushnir , S. Solouki , T. Sarig‐Meth , et al., “Systemic Inflammation and Autoimmunity in Women With Chronic Endometritis,” American Journal of Reproductive Immunology 75 (2016): 672–677. 26952510 10.1111/aji.12508

[21] D. J. Skarzynski , A. Z. Szóstek‐Mioduchowska , M. R. Rebordao , et al., “Neutrophils, Monocytes and Other Immune Components in the Equine Endometrium: Friends or Foes?,” Theriogenology 150 (2020): 150–157. 31973963 10.1016/j.theriogenology.2020.01.018

[22] R. H. Shih , C. Y. Wang , and C. M. Yang , “NF‐kappaB Signaling Pathways in Neurological Inflammation: A Mini Review,” Frontiers in Molecular Neuroscience 8 (2015): 77. 26733801 10.3389/fnmol.2015.00077 PMC4683208

[23] M. Zhou , Y. Yi , and L. Hong , “Oridonin Ameliorates Lipopolysaccharide‐Induced Endometritis in Mice via Inhibition of the TLR‐4/NF‐kappaB pathway,” Inflammation 42 (2019): 81–90. 30132202 10.1007/s10753-018-0874-8

[24] J. Liu , R. Kang , and D. Tang , “Signaling Pathways and Defense Mechanisms of Ferroptosis,” FEBS Journal 289 (2022): 7038–7050. 34092035 10.1111/febs.16059

[25] F. Ursini and M. Maiorino , “Lipid Peroxidation and Ferroptosis: The Role of GSH and GPx4,” Free Radical Biology & Medicine 152 (2020): 175–185. 32165281 10.1016/j.freeradbiomed.2020.02.027

[26] E. M. Corteselli , E. Gibbs‐Flournoy , S. O. Simmons , P. Bromberg , A. Gold , and J. M. Samet , “Long Chain Lipid Hydroperoxides Increase the Glutathione Redox Potential Through Glutathione Peroxidase 4,” Biochimica et Biophysica Acta‐General Subjects 1863 (2019): 950–959. 30844486 10.1016/j.bbagen.2019.03.002 PMC6823641

[27] Y. Huang , H. Wu , Y. Hu , et al., “Puerarin Attenuates Oxidative Stress and Ferroptosis via AMPK/PGC1alpha/Nrf2 Pathway After Subarachnoid Hemorrhage in Rats,” Antioxidants (Basel) 11 (2022): 1259. 35883750 10.3390/antiox11071259 PMC9312059

